# Pancreatic microbiota composition and diversity in pancreatitis-associated infection: a systematic review with focus on infected pancreatic necrosis

**DOI:** 10.3389/fmicb.2026.1916175

**Published:** 2026-09-17

**Authors:** Bo Wu, Wei Wei, Kaichen Zhang, Song Zhang

**Affiliations:** 1 Department of Intensive Care Medicine, Anyue County People's Hospital, Ziyang, Sichuan, China; 2 Department of Intensive Care Medicine, Affiliated Hospital of Chengdu University of Traditional Chinese Medicine, Chengdu, Sichuan, China

**Keywords:** acute pancreatitis, infected pancreatic necrosis, metagenomic next-generation sequencing, microbial diversity, pancreatic microbiota, pancreatitis-associated infection

## Abstract

Pancreatitis-associated intra-abdominal infection (PA-IAI), particularly infected pancreatic necrosis (IPN), is a serious complication of severe acute pancreatitis and contributes substantially to late morbidity and mortality. Sequencing-based technologies have expanded the detection of microorganisms in pancreatic infection; however, the characteristics of pancreatic microbiota and their clinical relevance have not been systematically summarized. A systematic literature search was conducted in seven electronic databases to identify studies evaluating pancreatic microbiota in patients with pancreatitis-associated infection. Studies reporting microbial composition or diversity using sequencing-based approaches were included. Study characteristics, microbial findings, diversity patterns, clinical outcomes, and methodological details were systematically extracted and synthesized. Six studies involving 235 participants were included; 188 participants contributed pancreatic or peripancreatic microbiota data. Most available evidence was derived from patients with IPN. Sequencing-based approaches generally detected a broader range of microbial taxa than conventional culture, with enteric-associated microorganisms frequently detected, while phylum-level dominance varied among studies. Reported diversity patterns varied between studies and could not be directly compared because of methodological heterogeneity. Although IPN was associated with worse clinical outcomes, current evidence did not demonstrate that specific microbial taxa or microbiota characteristics independently predicted mortality, organ failure, or treatment response. Current evidence indicates that pancreatic microbiota in pancreatitis-associated infection, particularly IPN, are characterized by frequent detection of enteric-associated microorganisms and substantial methodological heterogeneity. Sequencing-based approaches may provide additional microbiological information beyond conventional culture and may influence clinical management; however, whether sequencing-guided management changes improve antimicrobial stewardship or patient-centered outcomes remains uncertain. Future prospective studies using standardized microbiome workflows are required to determine the clinical significance of pancreatic microbial characteristics.

**Systematic review registration:**

https://www.crd.york.ac.uk/PROSPERO/view/CRD420261295285, identifier: CRD420261295285.

## 1 Introduction

Acute pancreatitis (AP) is among the most common gastrointestinal emergencies worldwide, and its incidence continues to increase globally. Although most patients experience a mild and self-limiting disease course, approximately 15%−20% develop severe acute pancreatitis (SAP), which is frequently complicated by infected pancreatic necrosis (IPN), sepsis, and multiple organ dysfunction syndrome. Once infection develops within necrotic pancreatic tissue, mortality increases substantially (Liu et al., 2025). Accumulating evidence suggests that infectious complications are not merely secondary events in AP but may actively contribute to disease progression and late-phase clinical deterioration. In particular, intra-abdominal and peripancreatic infections have emerged as major determinants of adverse outcomes in patients with SAP (Boxhoorn et al., 2020; van den Berg et al., 2021; Zhang X. et al., 2025).

Traditionally, the pancreas was considered a sterile organ. Advances in sequencing-based approaches, however, have challenged this concept. High-throughput approaches such as 16S rRNA sequencing and metagenomic next-generation sequencing (mNGS) have identified complex pancreatic and peripancreatic microbiota under pathological conditions (Thomas and Jobin, 2020; Li et al., 2024). These findings have renewed interest in the role of the gut-pancreas axis in pancreatic inflammation and infection.

Disruption of intestinal barrier integrity may contribute to this process. Intestinal ischemia, dysmotility, immune dysfunction, and increased mucosal permeability during AP may facilitate translocation of intestinal microorganisms and microbial products into the pancreas and surrounding tissues (Li et al., 2020; Wang et al., 2022; Ammer-Herrmenau et al., 2024). Several studies have reported depletion of beneficial commensals, including *Bifidobacterium* and *Faecalibacterium*, together with enrichment of opportunistic pathogens such as *Escherichia coli, Acinetobacter baumannii*, and *Enterococcus* species (Liu et al., 2024; Lupu et al., 2024). These microbial alterations may further exacerbate systemic inflammation through lipopolysaccharide-mediated signaling, impaired short-chain fatty acid production, and dysregulated immune responses (Pan et al., 2019; Yan et al., 2023).

Recent clinical investigations have also shown that intestinal microbial profiles correlate with disease severity, organ failure, and mortality in AP (Patel et al., 2021; Ammer-Herrmenau et al., 2024). Importantly, high similarity between intestinal and pancreatic microbiota profiles in patients with IPN provides indirect evidence for a possible contribution of gut-derived microorganisms to pancreatic infection (Lupu et al., 2024).

At the same time, widespread empirical antibiotic use in severe AP has raised concerns regarding antimicrobial resistance and microbiota disruption (Tenner et al., 2024; IAP/APA/EPC/IPC/JPS Working Group, 2025). A more precise understanding of pancreatic microbial composition may therefore have important implications for pathogen-directed therapy and individualized antimicrobial strategies.

Against this background, the present systematic review aimed to summarize current evidence regarding microbial composition and diversity in PA-IAI, with the available evidence largely derived from patients with IPN. Particular attention was given to sequencing-based microbiota studies and the potential role of gut microbial translocation in the pathogenesis of IPN.

## 2 Materials and methods

### 2.1 Study design

This systematic review was conducted in accordance with the Preferred Reporting Items for Systematic Reviews and Meta-Analyses (PRISMA) statement and according to a prospectively registered protocol in PROSPERO (CRD420261295285).

### 2.2 Search strategy

A comprehensive literature search was performed in PubMed, Embase, Web of Science, the Cochrane Library, China National Knowledge Infrastructure (CNKI), Wanfang Database, and VIP Database from database inception to February 1, 2026.

Search terms were developed using combinations of Medical Subject Headings (MeSH) and free-text keywords related to acute pancreatitis, IPN, microbiota, microbiome, intra-abdominal infection, and microbial sequencing. The detailed search strategy is provided in the Supplementary material.

### 2.3 Eligibility criteria

Studies were considered eligible if they met the following criteria:

1. Included adult patients with PA-IAI. Although the search strategy used the broader term “PA-IAI”, most eligible studies involved IPN or suspected IPN. Therefore, the findings of this review primarily reflect microbial characteristics associated with IPN rather than all possible infectious complications after pancreatitis;
2. Collected pancreatic necrotic tissue, peripancreatic fluid, or intra-abdominal effusion samples for microbial analysis;
3. Applied mNGS or other sequencing-based approaches for microbiota characterization;
4. Used observational or interventional study designs with extractable baseline microbiological data.

Reviews, case reports, conference abstracts, animal studies, duplicate publications, and studies lacking sufficient microbiological information were excluded.

### 2.4 Study selection

All retrieved records were imported into EndNote 21 for reference management and duplicate removal. Two investigators independently screened titles and abstracts, followed by full-text review of potentially eligible studies. Disagreements were resolved through discussion with a third reviewer. Reference lists of included studies and relevant reviews were manually screened to identify additional eligible studies.

### 2.5 Data extraction

Two investigators independently extracted study characteristics using a predefined data collection form. Extracted information included the overall study population, participants contributing pancreatic or peripancreatic microbiota data, confirmed IPN and sterile pancreatic necrosis (SPN) cases, historical controls, sample source, antibiotic exposure, sequencing methodology, microbial composition, microbial diversity, sequencing platforms, DNA extraction methods, bioinformatics pipelines, and clinical outcomes.

For studies including comparison groups that did not contribute microbiological samples, such as historical controls, these participants were considered part of the clinical study population but were not counted as microbiota analysis participants.

### 2.6 Data extraction of microbial diversity measures

Microbial diversity measures reported in the included studies were extracted directly from the original publications and their Supplementary materials. Reported alpha-diversity measures, including Shannon, Simpson, Chao1, ACE, and Sobs, as well as beta-diversity analyses such as Bray-Curtis dissimilarity, PCoA, and PLS-DA, were summarized descriptively when available.

To minimize potential bias arising from differences in sequencing depth, specimen processing, taxonomic classification, and bioinformatic pipelines, no alpha- or beta-diversity indices were recalculated by the review authors. Diversity measures were therefore interpreted only within the methodological context of the individual studies and were not quantitatively compared across studies.

### 2.7 Risk of bias assessment

A microbiota-specific risk-of-bias assessment tool was not applied because no universally accepted framework has been established for pancreatic microbiota studies. The risk of bias of included cohort studies was assessed using the Newcastle-Ottawa Scale (NOS). For the prospective single-arm study that specifically evaluated the diagnostic performance of mNGS-based microbial detection, the Quality Assessment of Diagnostic Accuracy Studies-2 (QUADAS-2) framework was additionally applied.

Because QUADAS-2 was not originally designed for microbiome sequencing studies, the Index Test domain was operationally adapted by adding microbiome-specific signaling questions addressing the reporting of sequencing platforms, DNA extraction methods, bioinformatic pipelines, predefined positivity criteria, contamination control procedures, and blinding of laboratory or bioinformatic personnel. Each signaling question was answered as Yes, No, or Unclear. Domain-level risk-of-bias judgments were based on the pattern of responses and the methodological information reported in the original study, with particular consideration given to whether incomplete reporting could plausibly introduce bias in microbial detection or interpretation.

These microbiome-specific questions were used as additional signaling questions within the QUADAS-2 Index Test domain and were not treated as a separately validated scoring system. Detailed signaling-question responses, domain-level judgments, and the rationale for each judgment are provided in Supplementary Table S1. The adapted QUADAS-2 assessment was used descriptively to identify potential sources of bias and methodological limitations rather than to generate an overall quality score. Any discrepancies between reviewers were resolved through consensus.

Ethics Statement: All included original studies reported obtaining ethics approval from the relevant institutional review boards and complied with applicable bioethical regulations for human research.

## 3 Results

### 3.1 Study selection

The database search identified 2,042 records. After removal of duplicates and stepwise screening of titles, abstracts, and full texts, six studies met the eligibility criteria and were included in the final analysis. The study selection process was performed according to the Preferred Reporting Items for Systematic Reviews and Meta-Analyses (PRISMA) 2020 guidelines. The numbers of identified, screened, assessed, and included studies are presented in the PRISMA 2020 flow diagram (Figure 1).

**Figure 1:**
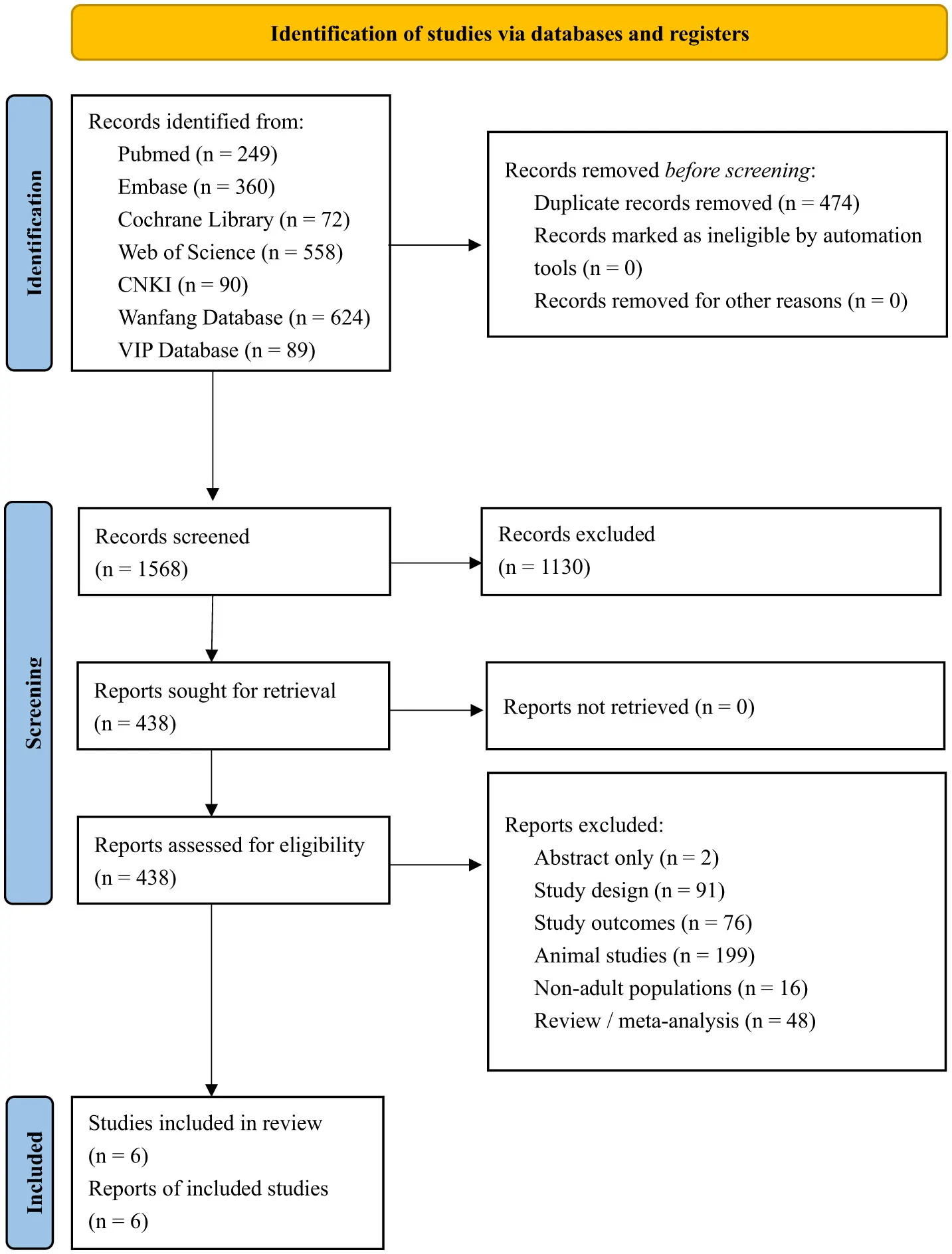
PRISMA flow diagram of literature screening.

### 3.2 Study characteristics

The six included studies enrolled 235 participants overall. Of these, 188 participants contributed pancreatic or peripancreatic microbiota data, including 129 confirmed IPN cases and 59 SPN cases. Hong et al. (2024) enrolled 20 patients with suspected IPN, of whom 17 had confirmatory IPN and three had SPN; these 20 patients contributed microbiota data, while the 40 historical controls were used only for clinical comparison. Lin et al. (2022) enrolled 42 patients overall; 35 participants were included in the microbiota-focused analysis (21 IPN and 14 SPN), whereas the remaining enrolled patients did not contribute to this microbiota synthesis. Five studies used cohort designs, whereas one was a prospective single-arm study. The characteristics of the six included studies are summarized in Table 1.

**Table 1:** Basic characteristics of included studies.

References	Design	Overall, *n*	Microbiota Participants, *n*	IPN, *n*	SPN, *n*	Other controls, *n*	Age (years), mean	Time of sampling	Antibiotic exposure	Risk of bias
Jiang (2020)	Prospective cohort study	5	5	5	0	5 (self-control)	39.2 ± 11.78	All patients were repeatedly punctured and cultured.	Yes	Low
Lin et al. (2022)	Prospective cohort study	42	35^Δ1^	21	14	0	44.1 ± 13.1	During the first surgical intervention	Yes	Low
Hong et al. (2023)	Retrospective cohort study	40	40	25	15	0	42 (31, 57)	The first line of CT-guided fine needle puncture	Yes	Low
Xu (2023)	Prospective cohort study	22	22	16	6	0	47.1 ± 11.2	AP patients with first minimally invasive intervention of pancreatic necrosis	Yes; 13 patients had received antibiotics.	Low
Hong et al. (2023)	Prospective single-arm study	60	20	17	3	40 (historical control) ^Δ2^	50 (34.5, 59.5)	CT-guided FNA was performed within 24 h of suspected IPN.	Yes	Some concerns^*^
Zhang P. et al. (2025)	Retrospective cohort study	66	66	45	21	0	48.8 ± 15.9	The first CT-guided fine needle puncture or percutaneous catheter drainage was performed.	Yes; 48 patients had received antibiotics	Low

IPN, infected pancreatic necrosis; SPN, sterile pancreatic necrosis; AP, acute pancreatitis; CT, computed tomography; FNA, fine needle aspiration.

^*^: Risk-of-bias assessment was performed using the adapted QUADAS-2 framework, with detailed signaling-question responses, domain-level judgments, and rationales provided in Supplementary Table S1.

Δ1: Seven patients died prior to surgical intervention, with no specimens obtained.

Δ2: The 40 historical controls were included for clinical outcome comparison only and did not contribute pancreatic microbiota sequencing data.

Most studies compared patients with IPN and SPN. Sample sources included necrotic pancreatic tissue, peripancreatic fluid collections, and intra-abdominal effusions. Four studies used mNGS-based approaches, while the remaining studies employed high-throughput amplicon sequencing.

Overall, substantial heterogeneity was observed across studies with respect to sequencing methodology, sample source, and microbiological analysis workflow.

### 3.3 Risk of bias assessment

Five cohort studies were assessed using the NOS. All included cohort studies were judged to have a low risk of bias according to the NOS, although limitations related to small sample size and single-center design were noted.

The prospective single-arm diagnostic study was assessed using the adapted QUADAS-2 framework. Domain-level assessment showed low risk of bias in the patient selection and flow and timing domains. Some concerns were identified in the Index Test domain because, although the sequencing platform, DNA extraction procedures, and bioinformatic workflow were reported, information regarding predefined positivity criteria, contamination controls, and blinding of laboratory or bioinformatic personnel was insufficient. Some concerns were also identified in the Reference Standard domain because information regarding blinding during interpretation was limited. Applicability concerns were generally low, although some limitations remained regarding the generalizability of sequencing-based methodologies. Detailed signaling-question responses, domain-level judgments, and the rationale for these judgments are presented in Supplementary Table S1. The adapted microbiome-specific signaling questions were used descriptively and were not treated as a separately validated scoring system.

Given the methodological heterogeneity and limited number of studies, quantitative pooling was not considered appropriate.

### 3.4 Microbiota composition of pancreatic and intra-abdominal samples

All six studies reported information on bacterial community composition (Table 2). Among the sequencing-based microbiota studies, one investigated microbiota composition at the phylum and species levels and demonstrated that Proteobacteria was the predominant phylum in both the IPN and SPN groups. At the species level, the most abundant taxa included *Serratia marcescens, Ralstonia* spp., and *Escherichia coli*, with *E. coli* accounting for 15.7% of the total relative abundance (Xu, 2023). Another study focused on the genus level and identified *Staphylococcus* as the dominant genus in necrotic tissue samples (Jiang, 2020).

**Table 2:** Study characteristics and microbiological findings.

References	Type of samples	Sequencing platform	Sequencing type	DNA extraction	Bioinformatics pipeline	Taxonomy	Flora composition
Jiang (2020)	Pancreatic necrosis	HiSeq	16S rDNA	Not reported	Not reported	Genus/species	16S rDNA sequencing predominantly detected *Staphylococcus* and *Comamonas* at the genus level, while microbial culture identified *Staphylococcus* spp. and *Escherichia coli* as dominant species.
Lin et al. (2022)	Peripancreatic specimens	BGISEQ-50/MGISEQ-2000	mNGS	TIANamp Micro DNA	Burrows-wheeler alignment	Species	*Enterococcus faecalis* prevailed in both mNGS and culture, followed by *Acinetobacter baumannii* and *Klebsiella pneumoniae*.
Hong et al. (2023)	Pancreatic necrosis	Illumina NextSeq 550	mNGS	TIANGEN DP316 kit	Burrows-wheeler alignment	Species	*Enterococcus faecalis* predominated on mNGS, followed by *Acinetobacter baumannii* and *Escherichia coli*. Culture most frequently yielded *E. faecalis* and *A. baumannii*.
Xu (2023)	Pancreatic necrosis puncture fluid	Illumina MiSeq	16S rRNA	OMEGA Soil DNA Kit	QIIME2	Phylum/species	At phylum level, Proteobacteria dominated pancreatic microbiota in both IPN and SPN groups. At the species level, the dominant taxa in the IPN group were *Serratia marcescens, Ralstonia* spp., and *Escherichia coli*.
Hong et al. (2024)	Pancreatic necrosis	Illumina NextSeq 550	mNGS	TIANGEN DP316 kit	Burrows-wheeler alignment	Species	*Klebsiella pneumoniae* was predominant, followed by *Acinetobacter baumannii*, in both mNGS and microbial culture.
Zhang P. et al. (2025)	Peripancreatic effusion	Illumina NextSeq 550	mNGS	QIAamp DNA Micro Kit	SE75bp	Species	The most common bacteria detected by mNGS and microbial culture were *Escherichia coli*, followed by *Acinetobacter baumannii*.

mNGS, metagenomic next-generation sequencing; IPN, Infected Pancreatic Necrosis; SPN, Sterile Pancreatic Necrosis; QIIME, Quantitative Insights Into Microbial Ecology.

The remaining four studies primarily analyzed microbiota composition at the species level. Among these studies, *Escherichia coli* was frequently detected, along with *Acinetobacter baumannii, Enterococcus faecium, Enterococcus faecalis*, and *Klebsiella pneumoniae* (Lin et al., 2022; Hong et al., 2023, 2024; Zhang P. et al., 2025). In addition, five studies compared sequencing-based findings with conventional culture. The degree of concordance varied across studies and should not be generalized as uniformly high. For example, Hong et al. (2024) reported complete concordance in 14 of 20 patients, partial concordance in 2, and discordant results in 4. Jiang (2020) used 16S rDNA sequencing rather than mNGS, further underscoring the methodological differences among the included studies.

Overall, enteric-associated microorganisms were frequently detected in pancreatic and peripancreatic samples across the included studies, although the specific taxa and relative abundances varied among studies.

### 3.5 Diversity characteristics of the pancreatic microbiota

Microbial diversity analyses were reported in two sequencing-based studies included in this review, although substantial heterogeneity existed in diversity metrics, sequencing approaches, and comparison groups. The reported diversity measures included Shannon, Simpson, Chao1, ACE, and Sobs indices, while beta-diversity analysis based on Bray-Curtis distance was available in these studies. Detailed diversity-related findings extracted directly from the original publications are summarized in Supplementary Table S2.

The included studies reported diversity patterns within their respective study populations, but these findings were not directly comparable across studies. Jiang (2020) reported Sobs, Chao1, ACE, Shannon, Simpson, and Bray-Curtis analyses based on OTU abundance data, and observed partial overlap between pancreatic necrotic tissue and intestinal microbial communities. Xu (2023) reported Chao1, ACE, Shannon, and Simpson indices and reported differences in microbial community structure between the IPN and SPN groups based on beta-diversity analysis. The study also reported greater intestinal-pancreatic microbial overlap in IPN patients, including higher shared ASV proportions and closer Bray-Curtis distances. Each reported diversity measure and its study-specific interpretation are traceable to the original publication in Supplementary Table S2.

Although individual studies identified differences in diversity-related measures between specific groups, these findings were not consistent across studies. The substantial heterogeneity in diversity metrics, sequencing strategies, and analytical approaches limited direct comparison and prevented the identification of a common pancreatic microbial diversity pattern associated with IPN.

### 3.6 Relationship between pancreatic microbiota findings and clinical outcomes

Across the included studies, patients with IPN consistently demonstrated worse clinical outcomes compared with those with SPN. These outcomes included prolonged hospitalization, longer ICU stay, increased healthcare costs, higher rates of organ failure, and increased mortality. Several studies reported associations between IPN status and adverse outcomes. Zhang P. et al. (2025) observed longer hospitalization duration, prolonged ICU stay, and increased medical costs among patients with IPN compared with SPN. Lin et al. (2022) reported higher mortality and longer hospital stay in patients with IPN, while Xu (2023) identified increased systemic complications and organ dysfunction in the IPN group.

Evidence linking specific microbial characteristics to clinical outcomes remains limited. Although enteric-associated microorganisms, including *Escherichia coli, Klebsiella pneumoniae, Acinetobacter baumannii*, and *Enterococcus* species, were frequently detected in IPN, none of the included studies demonstrated that the presence or relative abundance of individual microbial taxa independently predicted mortality, organ failure, or treatment response.

Limited longitudinal evidence was available regarding temporal changes in pancreatic microbiota during treatment. Jiang (2020) reported dynamic changes in intestinal and pancreatic microbial profiles during minimally invasive treatment of IPN, suggesting a potential relationship between microbial evolution and disease recovery. However, this observation was based on a very small sample size and requires validation in larger prospective cohorts.

## 4 Discussion

### 4.1 Principal findings

The present systematic review summarizes current evidence regarding microbiota characteristics of PA-IAI, with the available literature predominantly representing IPN. Across the six included studies, sequencing-based analyses generally demonstrated frequent detection of enteric-associated microorganisms in pancreatic and peripancreatic samples. Proteobacteria was reported as the predominant bacterial phylum in one sequencing-based study, while other studies mainly reported dominant taxa at the genus or species level, including *Enterococcus, Klebsiella pneumoniae*, and *Escherichia coli*. Two included studies compared intestinal and pancreatic microbial profiles and suggested greater overlap between these microbial communities in patients with IPN (Jiang, 2020; Xu, 2023).

Importantly, although IPN was consistently associated with worse clinical outcomes, including prolonged hospitalization, increased organ dysfunction, and higher mortality compared with SPN, these findings primarily reflect the clinical burden of pancreatic infection itself. Current studies have not demonstrated that specific microbial taxa or microbiota characteristics independently predict mortality, organ failure, or treatment response. Therefore, pancreatic microbiota alterations should currently be considered associated features of IPN rather than established prognostic biomarkers.

The interpretation of these findings requires consideration of substantial methodological heterogeneity. Differences in specimen types, sequencing platforms, analytical pipelines, and clinical characteristics may influence microbial composition and diversity estimates. Consequently, current evidence provides an important foundation for understanding microbial patterns in IPN but does not yet support definitive conclusions regarding causality or clinical application.

### 4.2 Interpretation of microbial patterns identified in pancreatic infection

A recurring observation across the included studies was the frequent detection of enteric-associated microorganisms within pancreatic and peripancreatic samples. The frequent detection of *Escherichia coli, Klebsiella pneumoniae, Acinetobacter baumannii*, and *Enterococcus* species suggests that IPN-associated microbial communities commonly include opportunistic organisms that are also found in intestinal microbial communities.

The overlap between intestinal and pancreatic microbial profiles reported in two studies provides indirect evidence supporting a possible gut-pancreas microbial connection in IPN (Jiang, 2020; Xu, 2023). These findings are compatible with a possible contribution of intestinal microbial translocation to pancreatic colonization during severe pancreatic injury; however, the available evidence remains observational and does not establish the directionality of microbial transmission or confirm the intestine as the direct source of pancreatic infection.

Microbial alterations may represent a contributing factor in infection development, but they may also result from severe inflammation, impaired intestinal function, antibiotic exposure, nutritional interventions, and prolonged critical illness. Moreover, most included studies were observational and involved relatively small patient populations, limiting the ability to determine causal relationships between microbial changes and disease progression.

Therefore, current evidence supports an association between an intestinal-associated microbial signature and IPN but does not establish whether specific microbial alterations directly drive pancreatic injury or determine clinical outcomes.

### 4.3 Interpretation of sequencing-based microbial detection

When interpreting microbial diversity findings, it is important to distinguish sequencing-based community analysis from culture-based microbial profiling. Conventional culture primarily identifies cultivable microorganisms under specific laboratory conditions and therefore does not provide a comprehensive assessment of microbial community structure or ecological diversity. In contrast, sequencing-based approaches enable community-level characterization and allow the calculation of diversity-related indices. Accordingly, differences between these methodologies should be interpreted as differences in microbial detection and characterization capacity rather than direct evidence of differences in the underlying ecological diversity.

Within the included studies, sequencing-based approaches, particularly mNGS, substantially expanded the characterization of pancreatic microbial communities in patients with suspected IPN. Compared with conventional detection methods, mNGS generally identified a broader spectrum of microbial taxa, including low-abundance and potentially difficult-to-culture microorganisms. Individual studies reported differences in microbial diversity measures between predefined comparison groups (Jiang, 2020; Xu, 2023). However, these findings should be interpreted cautiously, as higher diversity estimates obtained through sequencing may partly reflect increased microbial detection breadth rather than true alterations in pancreatic microbial ecology.

However, the ability to detect additional microbial signals should not be interpreted as equivalent to identifying clinically relevant pathogens or improving antimicrobial management. Importantly, sequencing-based detection does not necessarily indicate active infection because microbial DNA may originate from viable organisms, non-viable microbial fragments, colonization, or environmental contamination. This limitation is particularly relevant for pancreatic specimens, which are generally considered low-biomass samples. Therefore, interpretation of mNGS results requires integration with clinical presentation, inflammatory markers, imaging findings, conventional culture results, and quantitative microbial information when available.

Limited evidence was available regarding the influence of mNGS results on subsequent clinical management. For example, in Hong et al. (2024) mNGS results led to management changes in 16 of 20 patients with suspected IPN; however, the appropriateness of these changes and their effects on antimicrobial stewardship or patient-centered outcomes were not established. Therefore, in patients with suspected IPN, particularly after antibiotic exposure, current evidence does not demonstrate that sequencing-guided antimicrobial adjustment leads to improved antimicrobial stewardship, shorter hospitalization, reduced organ failure, or decreased mortality.

Future studies should prospectively evaluate whether integrating sequencing results into clinical decision-making can provide measurable therapeutic benefits.

### 4.4 Methodological heterogeneity and microbiome-specific limitations

Substantial methodological heterogeneity was observed across the included studies, which represents a major challenge for interpreting microbiota findings. Differences existed in specimen types, including pancreatic necrotic tissue, peripancreatic fluid collections, drainage samples, and intestinal samples. These biological niches may differ considerably in microbial biomass, oxygen exposure, host inflammatory environment, and contamination susceptibility.

In addition, variations in sequencing platforms, DNA extraction methods, sequencing depth, bioinformatics pipelines, and taxonomic classification strategies may influence microbial detection and diversity estimates (Poulsen et al., 2022; Yang and Chen, 2022). These methodological differences may partly explain the inconsistent diversity findings across studies. Therefore, whether microbial diversity is consistently increased or decreased in IPN cannot currently be determined.

Another important consideration is the influence of clinical confounders. Prior antibiotic exposure, timing of sampling, nutritional support, severity of organ dysfunction, and therapeutic interventions may substantially alter microbial composition. Because most available studies lacked standardized reporting of these factors, their potential contribution to observed microbial differences cannot be fully excluded.

Although NOS and QUADAS-2 were applied in this review, these tools were not specifically developed for microbiome research. Important methodological domains, including contamination control, negative controls, sample processing procedures, and bioinformatics quality control, are not comprehensively captured by conventional risk-of-bias instruments. Future systematic evaluations of microbiota studies would benefit from validated assessment frameworks specifically designed for sequencing-based investigations.

## 5 Potential clinical implications and future perspectives

The findings of this review provide several potential implications for understanding and managing IPN; however, these implications should be interpreted as hypothesis-generating rather than practice-changing evidence.

First, the observed overlap between intestinal and pancreatic microbial communities supports a possible contribution of gut-derived microbial alterations to IPN (Jiang, 2020; Xu, 2023). The same phenomenon is also mentioned in other literature (Li et al., 2024; Lupu et al., 2024). These findings are consistent with the broader rationale for maintaining intestinal barrier function through established supportive strategies, including appropriate enteral nutrition and avoidance of unnecessary antibiotic exposure (Patel et al., 2021). However, whether interventions targeting intestinal microbiota can reduce pancreatic infection remains unclear.

Second, sequencing-based approaches may provide additional microbiological information beyond conventional culture and may influence clinical management. However, although management changes were reported in some patients, their appropriateness and effects on antimicrobial stewardship or patient-centered outcomes have not been established (Hong et al., 2024). Prospective interventional studies are required to establish the clinical utility of incorporating sequencing results into routine practice.

Third, microbial signatures may represent potential candidates for future biomarker development. Although the frequent detection of opportunistic microorganisms and alterations in microbial diversity have been associated with IPN, current evidence is insufficient to determine whether microbiota characteristics provide prognostic information beyond established clinical indicators such as organ failure severity and disease extent.

Future research should focus on multicenter prospective cohorts with standardized sampling procedures, harmonized sequencing workflows, longitudinal microbiota monitoring, and integration of clinical, metabolomic, and immunological data. Such studies are needed to clarify the biological significance of pancreatic microbial alterations and determine whether microbiota-based approaches can ultimately improve diagnosis, treatment selection, or outcomes in severe pancreatitis.

## 6 Strengths and limitations

This systematic review provides a comprehensive synthesis of current sequencing-based evidence regarding microbiota composition and diversity in pancreatitis-associated infection. By integrating available mNGS and sequencing studies, this review summarizes current understanding of pancreatic microbial characteristics while highlighting important methodological challenges.

Nevertheless, several limitations should be acknowledged. First, most studies were observational cohort studies, including both prospective and retrospective designs, and were limited by small sample sizes and single-center settings, potentially introducing selection and information bias. Second, most studies focused on IPN, limiting the generalizability of the findings to other forms of PA-IAI. Third, currently available risk of bias assessment tools have limited applicability to microbiome research, as no dedicated instrument comprehensively evaluates methodological quality across key domains such as sample processing, DNA extraction, sequencing platforms, contamination control, and bioinformatics analysis. Consequently, risk-of-bias assessments may be subject to methodological inaccuracies (Goodrich et al., 2014; Nearing et al., 2021; Lampeter et al., 2023). In addition, confounding factors including prior antibiotic exposure, nutritional support, timing of surgical intervention, and underlying comorbidities may substantially influence microbial community structure (Zwart et al., 2022; De Lucia et al., 2023; Severino et al., 2023; Pourali et al., 2024). Finally, current studies remain limited in their investigation of fungal, viral, and microbial metabolite alterations, highlighting the need for future multi-omics studies.

## 7 Conclusion

Based on limited observational evidence, pancreatic and peripancreatic microbiota in pancreatitis-associated infection, particularly IPN, are characterized by frequent detection of enteric-associated opportunistic microorganisms. Proteobacteria was reported as the predominant bacterial phylum in one sequencing-based study, although species- and genus-level findings varied across studies. Sequencing-based approaches, especially mNGS, may provide broader characterization of microbial genetic signals compared with conventional culture methods and may enhance microbiological assessment in selected clinical settings. However, whether these findings translate into improved patient management requires prospective validation.

The observed overlap between intestinal and pancreatic microbial profiles supports a potential contribution of gut-derived microbial alterations to IPN, although the directionality and mechanisms of microbial transmission remain to be established. Future multicenter prospective studies using standardized sampling procedures, harmonized sequencing workflows, and integrated multi-omics analyses are needed to clarify the biological mechanisms and clinical relevance of pancreatic microbiota alterations in severe acute pancreatitis.

## Data Availability

All extracted data used for this systematic review are provided in the article and Supplementary materials. The extracted review dataset, including study characteristics and microbiological findings derived from the included studies, is publicly available in the Mendeley Data repository, doi: 10.17632/f9x9gn5f63.1.

## Supplementary material

Supplementary Table S1 Risk of bias assessment of included studies using the NOS and adapted QUADAS-2 framework.

Supplementary Table S2 Microbial diversity measures reported in the included studies.

Supplementary Table S3 PRISMA checklist.

Supplementary Table S4 Literature retrieval strategy.

## References

[B1] Ammer-Herrmenau C. Antweiler K. L. Asendorf T. Beyer G. Buchholz S. M. Cameron S. (2024). Gut microbiota predicts severity and reveals novel metabolic signatures in acute pancreatitis. Gut 73, 485–495. doi: 10.1136/gutjnl-2023-330987 38129103 PMC10894816

[B2] Boxhoorn L. Voermans R. P. Bouwense S. A. Bruno M. J. Verdonk R. C. Boermeester M. A. (2020). Acute pancreatitis. Lancet 396, 726–734. doi: 10.1016/S0140-6736(20)31310-6 32891214

[B3] De Lucia S. S. Candelli M. Polito G. Maresca R. Mezza T. Schepis T. (2023). Nutrition in acute pancreatitis: from the old paradigm to the new evidence. Nutrients 15:1939. doi: 10.3390/nu15081939 PMC10144915 37111158

[B4] Goodrich J. K. Di Rienzi S. C. Poole A. C. Koren O. Walters W. A. Caporaso J. G. (2014). Conducting a microbiome study. Cell 158, 250–262. doi: 10.1016/j.cell.2014.06.037 25036628 PMC5074386

[B5] Hong D. Wang P. Chen Y. Zhang J. Jiang W. Ye B. (2023). Detection of potential pathogen in pancreatic fluid aspiration with metagenomic next-generation sequencing in patients with suspected infected pancreatic necrosis. Dig. Liver Dis. 55, 243–248. doi: 10.1016/j.dld.2022.07.014 35948458

[B6] Hong D. Wang P. Xu Y. Xu S. Yu L. Tong Z. (2024). Metagenomic next-generation sequencing-based fine-needle aspiration in patients with suspected infected pancreatic necrosis. Clin. Transl. Gastroenterol. 15:e00726. doi: 10.14309/ctg.0000000000000726 PMC11272294 38870091

[B7] IAP/APA/EPC/IPC/JPS Working Group (2025). International Association of Pancreatology revised guidelines on acute pancreatitis 2025: supported and endorsed by the American Pancreatic Association, European Pancreatic Club, Indian Pancreas Club, and Japan Pancreas Society. Pancreatology 25, 770–814. doi: 10.1016/j.pan.2025.04.020 40651900

[B8] Jiang X. (2020). Study on the correlation between abdominal microecology and intestinal microecology in acute pancreatitis (Master's thesis). Anhui Medical University, Hefei.

[B9] Lampeter T. Love C. Tang T. T. Marella A. S. Lee H. Y. Oganyan A. (2023). Risk of bias assessment tool for systematic review and meta-analysis of the gut microbiome. Gut Microbiome 4:e13. doi: 10.1017/gmb.2023.12 PMC11406368 39295908

[B10] Li X. He C. Zhu Y. Lu N. H. (2020). Role of gut microbiota on intestinal barrier function in acute pancreatitis. World J. Gastroenterol. 26, 2187–2193. doi: 10.3748/wjg.v26.i18.2187 PMC7235204 32476785

[B11] Li Y. Li J. Li S. Zhou S. Yang J. Xu K. (2024). Exploring the gut microbiota's crucial role in acute pancreatitis and the novel therapeutic potential of derived extracellular vesicles. Front. Pharmacol. 15:1437894. doi: 10.3389/fphar.2024.1437894 39130638 PMC11310017

[B12] Lin C. Bonsu A. A. F. K. Li J. Ning C. Chen L. Zhu S. (2022). Application of metagenomic next-generation sequencing for suspected infected pancreatic necrosis. Pancreatology 22, 864–870. doi: 10.1016/j.pan.2022.07.006 35864066

[B13] Liu J. Yan Q. Li S. Jiao J. Hao Y. Zhang G. (2024). Integrative metagenomic and metabolomic analyses reveal the potential of gut microbiota to exacerbate acute pancreatitis. NPJ Biofilms Microbiomes 10:29. doi: 10.1038/s41522-024-00499-4 38514648 PMC10957925

[B14] Liu Q. Ruan K. An Z. Li L. Ding C. Xu D. (2025). Updated review of research on the role of the gut microbiota and microbiota-derived metabolites in acute pancreatitis progression and inflammation-targeted therapy. Int. J. Biol. Sci. 21, 1242–1258. doi: 10.7150/ijbs.108858 PMC11781165 39897025

[B15] Lupu V. V. Bratu R. M. Trandafir L. M. Bozomitu L. Paduraru G. Gimiga N. (2024). Exploring the microbial landscape: gut dysbiosis and therapeutic strategies in pancreatitis—a narrative review. Biomedicines 12:645. doi: 10.3390/biomedicines12030645 PMC10967871 38540258

[B16] Nearing J. T. Comeau A. M. Langille M. G. I. (2021). Identifying biases and their potential solutions in human microbiome studies. Microbiome 9:113. doi: 10.1186/s40168-021-01059-0 PMC8132403 34006335

[B17] Pan X. Fang X. Wang F. Li H. Niu W. Liang W. (2019). Butyrate ameliorates caerulein-induced acute pancreatitis and associated intestinal injury by tissue-specific mechanisms. Br. J. Pharmacol. 176, 4446–4461. doi: 10.1111/bph.14806 31347703 PMC6932943

[B18] Patel B. K. Patel K. H. Bhatia M. Iyer S. G. Madhavan K. Moochhala S. M. (2021). Gut microbiome in acute pancreatitis: a review based on current literature. World J. Gastroenterol. 27, 5019–5036. doi: 10.3748/wjg.v27.i30.5019 PMC8384740 34497432

[B19] Poulsen C. S. Ekstrøm C. T. Aarestrup F. M. Pamp S. J. (2022). Library preparation and sequencing platform introduce bias in metagenomic-based characterizations of microbiomes. Microbiol. Spectr. 10, e00090–e00022. doi: 10.1128/spectrum.00090-22 PMC9045301 35289669

[B20] Pourali G. Kazemi D. Chadeganipour A. S. Arastonejad M. Kashani S. N. Pourali R. (2024). Microbiome as a biomarker and therapeutic target in pancreatic cancer. BMC Microbiol. 24:16. doi: 10.1186/s12866-023-03166-4 38183010 PMC10768369

[B21] Severino A. Varca S. Airola C. Mezza T. Gasbarrini A. Franceschi F. (2023). Antibiotic utilization in acute pancreatitis: a narrative review. Antibiotics 12:1120. doi: 10.3390/antibiotics12071120 PMC10376815 37508216

[B22] Tenner S. Vege S. S. Sheth S. G. Sauer B. Yang A. Conwell D. L. (2024). American College of Gastroenterology guidelines: management of acute pancreatitis. Am. J. Gastroenterol. 119, 419–437. doi: 10.14309/ajg.0000000000002645 38857482 PMC13221274

[B23] Thomas R. M. Jobin C. (2020). Microbiota in pancreatic health and disease: the next frontier in microbiome research. Nat. Rev. Gastroenterol. Hepatol. 17, 53–64. doi: 10.1038/s41575-019-0242-7 31811279

[B24] van den Berg F. F. van Dalen D. Hyoju S. K. van Santvoort H. C. Besselink M. G. Wiersinga W. J. (2021). Western-type diet influences mortality from necrotising pancreatitis and demonstrates a central role for butyrate. Gut 70, 915–927. doi: 10.1136/gutjnl-2019-320430 PMC7917160 32873697

[B25] Wang Z. Liu J. Li F. Luo Y. Ge P. Zhang Y. (2022). The gut–lung axis in severe acute pancreatitis-associated lung injury: the protection by the gut microbiota through short-chain fatty acids. Pharmacol. Res. 182:106321. doi: 10.1016/j.phrs.2022.106321 35752356

[B26] Xu X. (2023). Study on the source of infection in infected pancreatic necrosis based on intestinal flora imbalance. (Master's thesis). Nanchang: Nanchang University.

[B27] Yan X. Li J. Wu D. (2023). The role of short-chain fatty acids in acute pancreatitis. Molecules 28:4985. doi: 10.3390/molecules28134985 PMC10343743 37446647

[B28] Yang L. Chen J. (2022). A comprehensive evaluation of microbial differential abundance analysis methods: current status and potential solutions. Microbiome 10:130. doi: 10.1186/s40168-022-01320-0 PMC9392415 35986393

[B29] Zhang P. Lu Z. Cao L. Fu L. Chen H. Sun Y. (2025). Diagnostic performance of pancreatic fluid aspiration through metagenomic next-generation sequencing for suspected infected pancreatic necrosis. Dig. Liver Dis. 57:401–407. doi: 10.1016/j.dld.2024.07.036 39155203

[B30] Zhang X. Zhu P. Zhang Y. Dai S. (2025). The impact of extra-pancreatic infections on outcomes of acute pancreatitis: a systematic review and meta-analysis. J. Hepatobiliary. Pancreat. Sci. 32, 350–359. doi: 10.1002/jhbp.12099 39950256

[B31] Zwart E. S. Jeleniewski S. Boyd L. N. C. Meijer L. L. Puik J. R. Zonderhuis B. M. (2022). Factors that influence the pancreatic and duodenal microbiome in patients undergoing pancreatic surgery. PLoS One 17:e0278377. doi: 10.1371/journal.pone.0278377 36525425 PMC9757549

