# Supplementary material for: Pancreatic microbiota composition and diversity in pancreatitis-associated infection: a systematic review with focus on infected pancreatic necrosis
Source: Front Microbiol. 2026 Sep 17;17:1916175. doi: 10.3389/fmicb.2026.1916175 (PMC13628582; doi:10.3389/fmicb.2026.1916175)
Supplement: Supplementary file 1 [file Table_2.docx]

| **Study** | **Source of microbial abundance data** | **Data format** | **Diversity measures reported** | **Reporting status** | **Key reported findings** |
| --- | --- | --- | --- | --- | --- |
| Jiang 2020 | original study Table 9 / 10 | OTU abundance | Sobs, Chao1, ACE, Shannon, Simpson, Bray-Curtis | Reported by the original study; no secondary calculation performed | Approximately 40% of OTUs were shared between pancreatic necrotic tissue and intestinal microbiota. PLS-DA showed partial overlap but distinct separation between the two microbial communities. |
| Xu 2023 | original study Table 5 / 6 / 7 | ASV abundance | Chao1, ACE, Shannon, Simpson, Bray-Curtis | Reported by the original study; no secondary calculation performed | There was a trend toward lower Chao1 and Shannon indices in the intestinal microbiota of the IPN group compared with the SPN group; however, the differences were not statistically significant (Kruskal–Wallis test, P > 0.05). IPN showed higher intestinal–pancreatic microbiota overlap than SPN (shared ASVs: 29.2% vs 10.3%), with closer Bray–Curtis distances (P = 0.003). |

**Supplementary Table S2.** Microbial diversity measures reported in the included studies

OUT: Operational Taxonomic Units; ASV: Amplicon Sequence Variant; ACE: Abundance-based Coverage Estimator; IPN: Infected Pancreatic Necrosis; SPN: Sterile Pancreatic Necrosis; PLS‑DA: Partial Least Squares Discriminant Analysis;

**Note:** Only microbial diversity measures directly reported in the original publications are summarized. No alpha- or beta-diversity indices were recalculated by the review authors. Studies that did not provide sufficient sample-level microbial abundance data were not subjected to secondary diversity calculations.
