# Supplementary material for: Pancreatic microbiota composition and diversity in pancreatitis-associated infection: a systematic review with focus on infected pancreatic necrosis
Source: Front Microbiol. 2026 Sep 17;17:1916175. doi: 10.3389/fmicb.2026.1916175 (PMC13628582; doi:10.3389/fmicb.2026.1916175)
Supplement: Supplementary file 3 [file Table_4.docx]

**Literature search strategy**

**Date:2026/2/1**

**PubMed**

| Search number | Query | Results |
| --- | --- | --- |
| #1 | "Pancreatitis"[MeSH] | 59866 |
| #2 | (((((((((((("pancreatiti*, Acute Edematous"[Title/Abstract])) OR ("Acute Edematous pancreatiti*"[Title/Abstract])) OR ("Edematous pancreatiti*, Acute"[Title/Abstract])) OR ("Pancreatic Parenchymal Edema*"[Title/Abstract])) OR ("Edema, Pancreatic Parenchymal"[Title/Abstract])) OR ("Parenchymal Edema, Pancreatic"[Title/Abstract])) OR ("Pancreatic Parenchyma with Edema"[Title/Abstract])) OR ("pancreatiti*, Acute"[Title/Abstract])) OR ("Acute pancreatiti*"[Title/Abstract])) OR ("Fat Necrosis, Peripancreatic"[Title/Abstract])) OR ("Necrosis, Peripancreatic Fat"[Title/Abstract])) OR ("Peripancreatic Fat Necros*"[Title/Abstract]) | 31671 |
| #3 | #1 OR #2 | 67466 |
| #4 | " Intraabdominal Infections"[Mesh] | 52196 |
| #5 | (((((((((((((((((((((("Infection*, Intraabdominal"[Title/Abstract])) OR ("Intraabdominal Infection*"[Title/Abstract])) OR ("Intra-Abdominal Infection*"[Title/Abstract])) OR ("Infection*, Intra-Abdominal"[Title/Abstract])) OR ("Intra Abdominal Infections"[Title/Abstract])) OR ("Abdominal infection*"[Title/Abstract])) OR ("Intraperitoneal infection*"[Title/Abstract])) OR ("Infected pancreatic necrosis"[Title/Abstract])) OR ("IPN"[Title/Abstract])) OR ("Pancreatic infected collection"[Title/Abstract])) OR ("Secondary infection of pancreatic necrosis"[Title/Abstract])) OR ("Abdominal Abscess"[MeSH Terms])) OR ("Abscess*, Intra-Abdominal"[Title/Abstract])) OR ("Intra-Abdominal Abscess*"[Title/Abstract])) OR ("Abscess, Intra Abdominal"[Title/Abstract])) OR ("Abscess*, Abdominal"[Title/Abstract])) OR ("Abdominal Abscess*"[Title/Abstract])) OR ("Peritonitis"[MeSH Terms])) OR ("Secondary Peritonitis"[Title/Abstract])) OR ("Peritonitis, Secondary"[Title/Abstract])) OR ("Primary Peritonitis"[Title/Abstract])) OR ("Peritonitis, Primary"[Title/Abstract]) | 52335 |
| #6 | #4 OR #5 | 73060 |
| #7 | "Microbiota"[MeSH] | 115060 |
| #8 | ((((((((((((((((((((((("Microbiotas"[Title/Abstract])) OR ("Microbial Communit*"[Title/Abstract])) OR ("Community, Microbial"[Title/Abstract])) OR ("microbial community compos*"[Title/Abstract])) OR ("Community Composition, Microbial"[Title/Abstract])) OR ("Community Structure, Microbial"[Title/Abstract])) OR ("Composition, Microbial Community"[Title/Abstract])) OR ("Microbial Community Structur*"[Title/Abstract])) OR ("microbiom*"[Title/Abstract])) OR ("Bacteria"[MeSH Terms])) OR ("Bacteri*"[Title/Abstract])) OR ("Eubacteria"[Title/Abstract])) OR ("Bacterial flora"[Title/Abstract])) OR ("Pathogen*"[Title/Abstract])) OR ("Fungi"[MeSH Terms])) OR ("Fungus"[Title/Abstract])) OR ("Fung*, Filamentous"[Title/Abstract])) OR ("Filamentous Fung*"[Title/Abstract])) OR ("Mold*"[Title/Abstract])) OR ("Mycobiota"[Title/Abstract])) | 3582867 |
| #9 | #7 OR #8 | 3601605 |
| #10 | #3 AND #6 AND #9 | 249 |

**Embase**

| Search number | Query | Results |
| --- | --- | --- |
| #1 | 'pancreatitis'/exp | 157837 |
| #2 | 'hereditary pancreatitis':ab,kw,ti OR 'pancreas inflammation':ab,kw,ti OR 'pancreatic inflammation':ab,kw,ti OR 'traumatic pancreatitis':ab,kw,ti OR 'pancreatitis':ab,kw,ti OR 'pancreatiti*, acute edematous':ab,kw,ti OR 'acute edematous pancreatiti*':ab,kw,ti OR 'edematous pancreatiti*, acute':ab,kw,ti OR 'pancreatic parenchymal edema*':ab,kw,ti OR 'edema, pancreatic parenchymal':ab,kw,ti OR 'parenchymal edema, pancreatic':ab,kw,ti OR 'pancreatic parenchyma with edema':ab,kw,ti OR 'pancreatiti*, acute':ab,kw,ti OR 'acute pancreatiti*':ab,kw,ti OR 'fat necrosis, peripancreatic':ab,kw,ti OR 'necrosis, peripancreatic fat':ab,kw,ti OR 'peripancreatic fat necros*':ab,kw,ti | 119353 |
| #3 | #1 OR #2 | 157956 |
| #4 | 'abdominal infection'/exp | 46309 |
| #5 | 'intra-abdominal infection*':ab,kw,ti OR 'infection*, intra-abdominal':ab,kw,ti OR 'intra abdominal infections':ab,kw,ti OR 'intraperitoneal infection*':ab,kw,ti OR 'abdomen infection':ab,kw,ti OR 'infection, abdomen':ab,kw,ti OR 'infection*, intraabdominal':ab,kw,ti OR 'intraabdominal infection':ab,kw,ti OR 'intraabdominal infections':ab,kw,ti OR 'abdominal infections':ab,kw,ti OR 'infected pancreatic necrosis':ab,kw,ti OR 'ipn':ab,kw,ti OR 'pancreatic infected collection':ab,kw,ti OR 'secondary infection of pancreatic necrosis':ab,kw,ti OR 'abdomen abscess':ab,kw,ti OR 'abdominal abscesses':ab,kw,ti OR 'abscess, abdomen':ab,kw,ti OR 'intra-abdominal abscess':ab,kw,ti OR 'intra-abdominal abscesses':ab,kw,ti OR 'intraabdominal abscess':ab,kw,ti OR 'intraabdominal abscesses':ab,kw,ti OR 'abdominal abscess':ab,kw,ti OR 'diffuse peritonitis':ab,kw,ti OR 'intraperitoneal infection':ab,kw,ti OR 'peritoneal infection':ab,kw,ti OR 'perivisceritis':ab,kw,ti OR 'secondary peritonitis':ab,kw,ti OR 'peritonitis':ab,kw,ti | 70795 |
| #6 | #4 OR #5 | 97798 |
| #7 | 'microflora'/exp | 283076 |
| #8 | 'microbial communit*':ab,kw,ti OR 'community, microbial':ab,kw,ti OR 'microbial community compos*':ab,kw,ti OR 'community composition, microbial':ab,kw,ti OR 'community structure, microbial':ab,kw,ti OR 'composition, microbial community':ab,kw,ti OR 'microbial community structur*':ab,kw,ti OR 'microbiom*':ab,kw,ti OR 'microbial flora':ab,kw,ti OR 'microbiota':ab,kw,ti OR 'bacteria':ab,kw,ti OR 'eubacteria':ab,kw,ti OR 'eubacterium (group)':ab,kw,ti OR 'eubacterium (imperium)':ab,kw,ti OR 'eubacterium (urkingdom)':ab,kw,ti OR 'schizomycetes':ab,kw,ti OR 'bacterium':ab,kw,ti OR 'eumycota':ab,kw,ti OR 'fungi':ab,kw,ti OR 'fungi, unclassified':ab,kw,ti OR 'fungus':ab,kw,ti OR 'mold*':ab,kw,ti OR 'mycobiota':ab,kw,ti OR 'metagenom*':ab,kw,ti OR '16s rrna':ab,kw,ti OR 'next-generation sequencing':ab,kw,ti OR 'pathogen*':ab,kw,ti | 2528269 |
| #9 | #7 OR #8 | 2578864 |
| #10 | #3 AND #6 AND #9 | 360 |

Cochrane

| Search number | Query | Results |
| --- | --- | --- |
| #1 | MeSH descriptor: [Pancreatitis] explode all trees | 1912 |
| #2 | (pancreatiti*, Acute Edematous):ti,ab,kw OR (Acute Edematous pancreatiti*):ti,ab,kw OR (Edematous pancreatiti*, Acute):ti,ab,kw OR (Pancreatic Parenchymal Edema*):ti,ab,kw OR (Edema, Pancreatic Parenchymal):ti,ab,kw OR (Parenchymal Edema, Pancreatic):ti,ab,kw OR (Pancreatic Parenchyma with Edema):ti,ab,kw OR (pancreatiti*, Acute):ti,ab,kw OR (Acute pancreatiti*):ti,ab,kw OR (Fat Necrosis, Peripancreatic):ti,ab,kw OR (Necrosis, Peripancreatic Fat):ti,ab,kw OR (Peripancreatic Fat Necros*):ti,ab,kw | 3265 |
| #3 | #1 OR #2 | 4020 |
| #4 | MeSH descriptor: [Intraabdominal Infections] explode all trees | 1703 |
| #5 | (Intraabdominal Infection*):ti,ab,kw OR (Infection*, Intraabdominal):ti,ab,kw OR (Intra-Abdominal Infection*):ti,ab,kw OR (Infection*, Intra-Abdominal):ti,ab,kw OR (Intra Abdominal Infections):ti,ab,kw OR (Abdominal infection*):ti,ab,kw OR (Intraperitoneal infection*):ti,ab,kw OR (Infected pancreatic necrosis):ti,ab,kw OR (IPN):ti,ab,kw OR (Pancreatic infected collection):ti,ab,kw OR (Secondary infection of pancreatic necrosis):ti,ab,kw OR MeSH descriptor: [Abdominal Abscess] explode all trees OR (Abscess*, Intra-Abdominal):ti,ab,kw OR (Intra-Abdominal Abscess*):ti,ab,kw OR (Abscess, Intra Abdominal):ti,ab,kw OR (Abscess*, Abdominal):ti,ab,kw OR (Abdominal Abscess*):ti,ab,kw OR MeSH descriptor: [Peritonitis] explode all trees OR (Secondary Peritonitis):ti,ab,kw OR (Peritonitis, Secondary):ti,ab,kw OR (Primary Peritonitis):ti,ab,kw OR (Peritonitis, Primary):ti,ab,kw | 11161 |
| #6 | #4 OR #5 | 11810 |
| #7 | MeSH descriptor: [Microbiota] explode all trees | 2797 |
| #8 | (Microbiotas):ti,ab,kw OR (Microbial Communit*):ti,ab,kw OR (Community, Microbial):ti,ab,kw OR (microbial community compos*):ti,ab,kw OR (Community Composition, Microbial):ti,ab,kw OR (Community Structure, Microbial):ti,ab,kw OR (Composition, Microbial Community):ti,ab,kw OR (Microbial Community Structur*):ti,ab,kw OR (microbiom*):ti,ab,kw OR MeSH descriptor: [Bacteria] explode all trees OR (Bacteri*):ti,ab,kw OR (Eubacteria):ti,ab,kw OR (Bacterial flora):ti,ab,kw OR (Pathogen*):ti,ab,kw OR MeSH descriptor: [Fungi] explode all trees OR (Fungus):ti,ab,kw OR (Fung*, Filamentous):ti,ab,kw OR (Filamentous Fung*):ti,ab,kw OR (Mold*):ti,ab,kw OR (Mycobiota):ti,ab,kw | 85221 |
| #9 | #7 OR #8 | 85272 |
| #10 | #3 AND #6 AND #9 | 72 |

Web of science

| Search number | Query | Results |
| --- | --- | --- |
| #1 | TS= (Pancreatitis OR pancreatiti*, Acute Edematous OR Acute Edematous pancreatiti* OR Edematous pancreatiti*, Acute OR Pancreatic Parenchymal Edema* OR Edema, Pancreatic Parenchymal OR Parenchymal Edema, Pancreatic OR Pancreatic Parenchyma with Edema OR pancreatiti*, Acute OR Acute pancreatiti* OR Fat Necrosis, Peripancreatic OR Necrosis, Peripancreatic Fat OR Peripancreatic Fat Necros*) | 72721 |
| #2 | TS= (Intraabdominal Infections OR Infection*, Intraabdominal OR Intraabdominal Infection* OR Intra-Abdominal Infection* OR Infection*, Intra-Abdominal OR Intra Abdominal Infections OR Abdominal infection* OR Intraperitoneal infection* OR Infected pancreatic necrosis OR IPN OR Pancreatic infected collection OR Secondary infection of pancreatic necrosis OR Abdominal Abscess OR Abscess*, Intra-Abdominal OR Intra-Abdominal Abscess* OR Abscess, Intra Abdominal OR Abscess*, Abdominal OR Abdominal Abscess* OR Peritonitis OR Secondary Peritonitis OR Peritonitis, Secondary OR Primary Peritonitis OR Peritonitis, Primary) | 88501 |
| #3 | TS= (Microbiota OR Microbiotas OR Microbial Communit* OR Community, Microbial OR microbial community compos* OR Community Composition, Microbial OR Community Structure, Microbial OR Composition, Microbial Community OR Microbial Community Structur* OR microbiom* OR Bacteria OR Bacteri* OR Eubacteria OR Bacterial flora OR Pathogen* OR Fungi OR Fungus OR Fung*, Filamentous OR Filamentous Fung* OR Mold* OR Mycobiota) | 3079253 |
| #5 | #1 AND #2 AND #3 | 558 |

China National Knowledge Infrastructure (CNKI)

| Search number | Query | Results |
| --- | --- | --- |
| #1 | SU=('胰腺炎' + '急性胰腺炎' + '慢性胰腺炎' + '重症急性胰腺炎' + '急性坏死性胰腺炎' + '急性水肿型胰腺炎' + '胰腺实质水肿' + '胰周脂肪坏死') | 66267 |
| #2 | SU=('腹腔感染' + '腹腔内感染' + '继发胰腺感染' + '感染性胰腺坏死' + '胰腺坏死继发感染' + '腹腔脓肿' + '腹腔内脓肿' + '腹膜炎' + '继发性腹膜炎' + '原发性腹膜炎') | 18370 |
| #3 | SU=('微生物组' + '微生物' + '微生物群落组成' + '微生物群落结构' + '病原菌' + '细菌' + '真细菌' + '真菌' + '丝状菌' + '霉菌') | 803139 |
| #5 | #1 AND #2 AND #3 | 90 |

Wanfang Database

| Search number | Query | Results |
| --- | --- | --- |
| #1 | 主题:(胰腺炎 OR 急性胰腺炎 OR 慢性胰腺炎 OR 重症急性胰腺炎 OR 急性坏死性胰腺炎 OR 急性水肿型胰腺炎 OR 胰腺实质水肿 OR 胰周脂肪坏死) | 140939 |
| #2 | 主题:(腹腔感染 OR 腹腔内感染 OR 继发胰腺感染 OR 感染性胰腺坏死 OR 胰腺坏死继发感染 OR 腹腔脓肿 OR 腹腔内脓肿 OR 腹膜炎 OR 继发性腹膜炎 OR 原发性腹膜炎) | 67304 |
| #3 | 主题:(微生物组 OR 微生物 OR 微生物群落组成 OR 微生物群落结构 OR 病原菌 OR 细菌 OR 真细菌 OR 真菌 OR 丝状菌 OR 霉菌) | 2076964 |
| #5 | #1 AND #2 AND #3 | 624 |

VIP Database

| Search number | Query | Results |
| --- | --- | --- |
| #1 | M=(胰腺炎+急性胰腺炎+慢性胰腺炎+重症急性胰腺炎+急性坏死性胰腺炎+急性水肿型胰腺炎+胰腺实质水肿+胰周脂肪坏死) | 57430 |
| #2 | M=(腹腔感染+腹腔内感染+继发胰腺感染+感染性胰腺坏死+胰腺坏死继发感染+腹腔脓肿+腹腔内脓肿+腹膜炎+继发性腹膜炎+原发性腹膜炎) | 14933 |
| #3 | M=(微生物组+微生物+微生物群落组成+微生物群落结构+病原菌+细菌+真细菌+真菌+丝状菌+霉菌) | 388316 |
| #5 | #1 AND #2 AND #3 | 89 |
